# Guided self-determination-young versus standard care in the treatment of young females with type 1 diabetes: study protocol for a multicentre randomized controlled trial

**DOI:** 10.1186/s13063-017-2296-6

**Published:** 2017-11-24

**Authors:** Josephine Haas, Martina Persson, Anna Lena Brorsson, Eva Hagström Toft, Anna Lindholm Olinder

**Affiliations:** 1 Department of Clinical Science and Education, Karolinska Institute, Södersjukhuset, Stockholm Sweden; 2 Sachs’ Children and Youth Hospital, Södersjukhuset, Stockholm Sweden; 3 Department of Medicine, Clinical Epidemiological Unit, Karolinska Institute, Stockholm, Sweden; 4 Department of Women’s and Children’s Health, Karolinska Institute and Hospital, Stockholm, Sweden; 5 Department of Medicine, Karolinska Institute at Karolinska University Hospital, Huddinge, Sweden; 6 Ersta Hospital, Diabetes Unit, Stockholm, Sweden; 7 Department of Medical Sciences, Uppsala University, Uppsala, Sweden

**Keywords:** Type 1 diabetes, Adolescents, Person-centred care, Guided self-determination-young (GSD-Y), Female, Intervention

## Abstract

**Background:**

Female adolescents with type 1 diabetes mellitus (T1DM) have the most unsatisfactory glycaemic control of all age groups and report higher disease burden, poorer perceived health, and lower quality of life than their male counterparts. Females with T1DM face an excess risk of all-cause mortality compared with men with T1DM. New methods are needed to help and support young females with T1DM to manage their disease.

A prerequisite for successful diabetes management is to offer individualized, person-centred care and support the patient’s own motivation. Guided self-determination (GSD) is a person-centred reflection and problem-solving method intended to support the patient’s own motivation in the daily care of her diabetes and help develop skills to manage difficulties in diabetes self-management. GSD has been shown to improve glycaemic control and decrease psychosocial stress in young women with T1DM. The method has been adapted for adolescents and their parents, termed GSD-young (GSD-Y). The aim of this study was to evaluate whether an intervention with GSD-Y in female adolescents with T1DM leads to improved glycaemic control, self-management, treatment satisfaction, perceived health and quality of life, fewer diabetes-related family conflicts, and improved psychosocial self-efficacy.

**Methods/design:**

This is a parallel-group randomized controlled superiority trial with an allocation ratio of 1:1. One hundred female adolescents with T1DM, 15–20 years of age, and their parents (if < 18 years of age), will be included. The intervention group will receive seven individual GSD-Y education visits over 3 to 6 months. The control group will receive standard care including regular visits to the diabetes clinic. The primary outcome is level of glycaemic control, measured as glycosylated haemoglobin (HbA1c). Secondary outcomes include diabetes self-management, treatment satisfaction, perceived health and quality of life, diabetes-related family conflicts, and psychosocial self-efficacy. Data will be collected before randomization and at 6 and 12 months.

**Discussion:**

Poor glycaemic control is common in female adolescents and young adults with T1DM. Long-standing hyperglycaemia increases the risks for severe complications and may also have an adverse impact on the outcome of future pregnancies. In this study, we want to evaluate if the GSD-Y method can be a useful tool in the treatment of female adolescents with T1DM.

**Trial registration:**

Current controlled trials, ISRCTN57528404. Registered on 18 February 2015.

**Electronic supplementary material:**

The online version of this article (doi:10.1186/s13063-017-2296-6) contains supplementary material, which is available to authorized users.

## Background

### Diabetes and female adolescents

It is well established that poor glycaemic control during adolescence and young adulthood increase the risks of acute and long-term diabetes-related complications [1]. Recommended treatment for children and adolescents with type I diabetes mellitus (T1DM) is multiple daily injections (MDI) or continuous subcutaneous insulin infusion (CSII) [2]. Intensive diabetes management has been in the clinical routine in Sweden for many years. The Swedish recommended level of glycaemic control, measured as glycosylated haemoglobin (HbA1c), for children and adolescents (<18 years) has recently changed (in 2017) from 57 mmol/mol or below to 48 mmol/mol or below without increasing the number of hypoglycaemic episodes [2]. For adults, the corresponding target is HbA1c < 52 mmol/mol [3]. To reach the recommended level it is necessary to measure blood glucose frequently or to use continuous or flash glucose monitoring (CGM/FGM). In Sweden, only around 55% of the paediatric population with T1DM reached the former HbA1c target of < 58 mmol/mol [4]. After starting school, at 7 years of age, HbA1c tends to increase with age and female adolescents have the most unsatisfactory glycaemic control of all age groups [4]. A similar pattern has also been found in other European countries [5]. Girls with T1DM report a higher disease burden, poorer perceived health, and lower quality of life (QoL) than boys [6–8], and females with T1DM face a 40% excess risk of all-cause mortality compared with men with T1DM [9]. Furthermore, women with T1DM have a significantly higher healthcare expenditure than men [10]. A nationwide study from Sweden demonstrated that female sex and high HbA1c adjacent to the diagnosis of T1DM are risk factors for poor control in young adults with increased prevalence of nephropathy and retinopathy [11]. This is in line with findings from previous studies reporting that female gender is a risk factor for microvascular complications [12–14]. Girls with T1DM have significantly higher mean body mass index (BMI) at follow-up compared with their male counterparts [15], and an elevated BMI is associated with higher levels of HbA1c [15]. Furthermore, high pre-pregnancy BMI is an important risk factor for adverse outcome in type 1 diabetic pregnancies [16]. In the report from the Public Health Agency of Sweden 2016, female adolescents (16–29 years) reported a higher degree of both mild and severe anxiety compared with their male counterparts [17]. In females with T1DM, but not in males, means of communication and a perception of being able to control the disease have been shown to be important contributing factors for achieved level of glycaemic control [18]. Furthermore, the coping strategy “ventilating negative feelings” was associated with poor metabolic control among females [18]. Active coping, in this case referring to how to rationally manage a problem, was found to be related to improved metabolic control in adolescents with T1DM [19]. On the other hand, emotion focused coping, such as behavioural disengagement and aggressive coping, was related to poor metabolic control [19]. This emphasizes the importance of finding out the patient’s coping strategies, views, and perceptions of her diabetes.

### Adolescence

During adolescence the needs of healthcare are distinctly different from the needs of younger children and adults. The patient’s need for liberation and self-determination often interferes with diabetes self-management [20] which makes diabetes treatment challenging [21]. Both treatment-related factors and factors related to the individual influence metabolic control. It is recommended to provide health education strategies that optimize self-care. This can involve problem solving, clarifying priorities and target setting, as well as using new technology [22]. Development of equipment for glucose monitoring and insulin delivery has significantly improved the outcome for patients with T1DM. However, adherence to a complex, demanding, and intensive diabetes regimen requires life-long changes in behaviour and involves daily performances of several unpleasant tasks. In the aspect of QoL, it may be just as important to meet the adolescent’s developmental needs as any other diabetes-specific treatment [20, 23].

Adolescents with chronic conditions have been found to have fewer protective factors and more risky behaviour compared to their healthy peers [24]. Family support is an important protective factor in reducing risky behaviour [24]. Communication within families and maintenance of parental support have been shown to contribute to metabolic control in adolescents with T1DM [22, 25, 26], but the patient’s own commitment is crucial for successful management. It is more common that children have high HbA1c in families who have many diabetes-related conflicts [27]. Healthcare professionals should support adolescents and parents to find both new means for parental involvement and levels of communication that suits both the adolescent and the parents [23, 28].

Finding out what is unique for the patient in a person-centred approach and in aspects of developmental, individual, and family factors is of great importance.

### Guided self-determination-young

Person-centred care aims at engaging the person as an active partner in his/her own care and treatment and at supporting the patient’s own ability to manage the disease in a positive way [29]. Guided self-determination (GSD) is an example of an empowerment-based education method providing person-centred care. By using worksheets for reflection and problem-solving, GSD intends to guide the patient to become self-determined and to obtain skills for better diabetes self-management [30]. GSD has been demonstrated to improve glycaemic control and decrease psychosocial stress in young adult women (18–35 years) with T1DM [31]. The method has been adapted to adolescents, termed guided self-determination-young (GSD-Y) [32–34], but data on its effect on glycaemic control and other outcomes in this age group are scarce [35]. There is only one study of limited size (*n* = 57) investigating the effect of GSD-Y in girls and boys with T1DM (aged 13–18 years) [32]. In that study, no significant reduction in HbA1c was demonstrated but the intervention improved the patient’s motivation for diabetes self-management [32]. Preliminary results from an ongoing study on adolescents aged 12–18 years starting treatment with CSII [34, 35] show decreased diabetes-related family conflicts and a larger decrease in HbA1c in the intervention group 6 months after intervention with the GSD-Y method.

It is clear that we need to find new methods to help and support young females with T1DM to manage their disease, and previous studies with the GSD method have shown positive results, especially for young adult females. Thus, the GSD method is a promising tool.

### Study aim and hypothesis

The aim of this study is to evaluate if an intervention with GSD-Y, in female adolescents and their parents, leads to improved glycaemic control, self-management, treatment satisfaction, perceived health and QoL, fewer diabetes-related family conflicts, and improved psychosocial self-efficacy.

Our hypothesis is that the GSD-Y method can improve glycaemic control, perceived health, and QoL among young females with T1DM.

## Methods/design

### Study design and setting

**Fig. 1:**
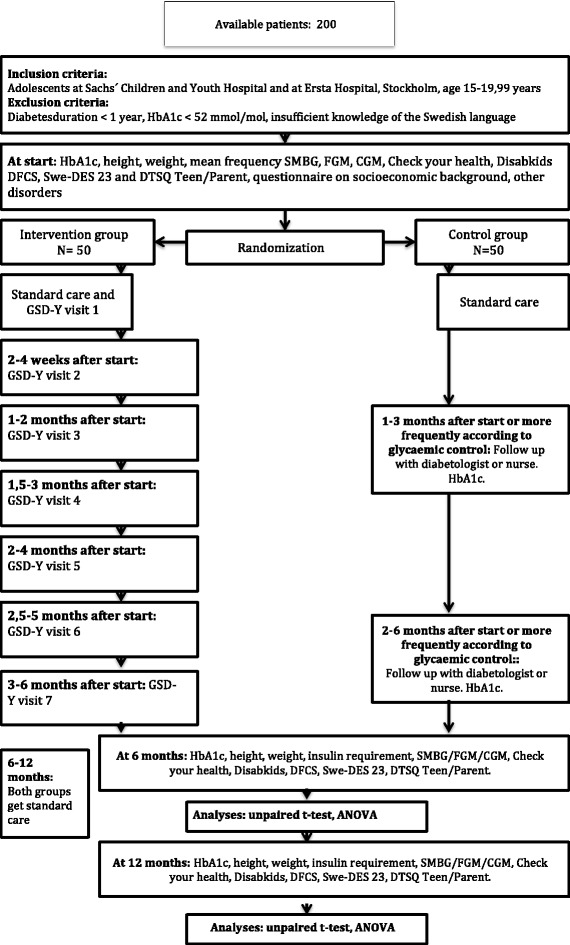
Flow chart for GSD-Y versus standard care. *ANOVA* analysis of variance, *CGM* continuous glucose monitoring, *DFCS* Diabetes Family Conflict Scale, *DTSQ* Diabetes Treatment Satisfaction Questionnaire, *FGM* flash glucose monitoring, *GSD*-*Y* guided self-determination-young, *HbA1c* glycosylated haemoglobin, *SMBG* self-monitoring of blood glucose, *Swe*-*DES 23* Swedish Diabetes Empowerment Scale

This is a parallel-group multicentre randomized controlled trial with an allocation ratio of 1:1 and a superiority framework. The study will be conducted at the Unit for Youth at Sachs’ Children and Youth Hospital (aged 15–20 years old) and at the outpatient diabetes clinic at Ersta Hospital (aged 18–20 years old) in Stockholm, Sweden. A flow chart of the study is presented in Fig. 1. The GSD-Y method was tested in six patients at our clinic before the study started and the questionnaires that we use are all validated and have been used by our research group in previous studies.

**Fig. 2:**
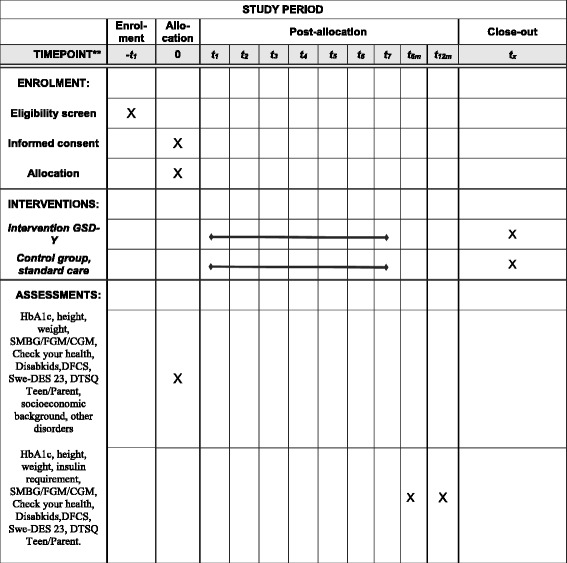
SPIRIT figure. *CGM* continuous glucose monitoring, *DFCS* Diabetes Family Conflict Scale, *DTSQ* Diabetes Treatment Satisfaction Questionnaire, *FGM* flash glucose monitoring, *GSD*-*Y* guided self-determination-young, *HbA1c* glycosylated haemoglobin, *SMBG* self-monitoring of blood glucose, *Swe*-*DES 23* Swedish Diabetes Empowerment Scale

This paper follows the Standard Protocol Items Recommendations for Interventional Trials (SPIRIT) guidelines for intervention trials. The SPIRIT checklist and figure are included as Additional file 1 and Fig. 2.

### Participants

One hundred female adolescents with T1DM, aged 15–20 years, with parents (if participants are under 18 years of age) will be included. Exclusion criteria includes a diagnosis of diabetes within the past year, or HbA1c below the national recommended level for adults (< 52 mmol/mol) and if the adolescent or their parents have difficulties understanding Swedish. The usual healthcare provider (HCP) at the diabetes outpatient clinic, supervised by JH, will enrol participants in the study. In Sweden, patients with T1DM are only treated at specialist centres and no concomitant care is involved in the diabetes treatment. Written informed consent for participation in the study will be obtained from adolescents and their parents (if participants are under 18 years of age) and given to the HCP and then collected by JH or EHT and stored in a locked and fireproof cabinet. Participants will be randomized to either the intervention or control group after filling in the written informed consent and the baseline questionnaires.

### Randomization

Participants are randomized using opaque sealed envelopes containing a once-folded piece of paper with written information on which group the participant is randomized to: intervention or control group. The envelopes are prepared by two researchers not involved in the study using coin tossing. The researchers will toss a coin and then, according to either “heads” or “tails”, put a once-folded piece of paper, with written information on either intervention or control group, in an opaque envelope and seal it. This coin-tossing will continue in blocks of two, i.e. one out of the two first envelopes will contain a paper that says “intervention” and the other envelope will contain a paper that says “control”. The first two envelopes will then be marked by “1” and “2” but it will be unknown which one contains which group. The coin-tossing continues until 100 envelopes are prepared and marked with the numbers “1” to “100”. At each randomization, the envelopes will be randomly mixed in groups of three with the number on the envelope face down, and the participant will choose one of these envelopes herself.

The structure of the GSD-Y method does not allow further blinding of group allocation after the randomization process.

### Intervention

Individuals in the intervention group will receive seven individual GSD-Y visits (of 1–1.5 h) with a facilitator (either a diabetes nurse or a physician) over 3 to 6 months. The facilitator is educated in the GSD-Y method. The GSD-Y method includes that the participant works with structured reflection sheets. By filling in reflection sheets before each visit and using their own words and drawings, participants systematically explore and express their own experiences and difficulties with diabetes in daily life. In addition, if the participant is under 18 years of age, her parents will be offered one individual visit and one visit together with the participant according to the GSD-Y method. The topics for the different visits are presented in Table 1. The first reflection sheet will be sent or given to the participants before the first visit. For subsequent visits, the reflection sheet for the next meeting will be distributed at that visit and completed before the next visit. In the dialogue between the participant and the facilitator, the facilitator uses different communication methods including mirroring, active listening, and value-clarifying responses. Those participants who have taken part in the first five GSD-Y visits will be considered to have completed the education. Visits are booked in consultation with each participant and, if a participant misses out of a visit, she will be offered another appointment provided she wants to continue with the study.

**Table 1:** Overview of reflections sheets

Visit 1	Your life with diabetes from the beginning to now
	Written invitation to work together in a new way
	Two ways of looking at glycosylated haemoglobin (HbA1c)
	Agreement on things to work on
Visit 2	Your life with diabetes from the beginning to now
	Important events and periods in your life
	What do you find difficult at present living with your diabetes?
	Your plans for changing your way of life
Visit 3	Values and opportunities
	Unfinished sentences: needs, values, experiences, and opportunities
	Individual visits for parents are offered
Visit 4	Diabetes in your life
	A picture or expression describing your life with diabetes
	Room for diabetes in your life
	Shared responsibility between adolescent and parent for diabetes in daily life
	Common name for difficulty in your life with diabetes
	Agreement on things to work on until next visit
Visit 5	Problem identification and problem solving
	Current problem solving
	Dynamic problem solving
	Agreement on things to work on until next visit
Visit 6	Different ways of looking at numbers
	Blood glucose (BG) tests and your reasons for checking
	Actual BG numbers and wishes
	Your plan for BG regulation in the short and long run
	Common name for a difficulty in your life with diabetes
	Agreement on things to work on until next visit
Visit 7	Problem identification and problem solving
	Current problem solving
	Dynamic problem solving
	Solved problems and subjects to continue working on
	Visit along with parents is offered

The control group will receive standard care, which includes visits to the diabetes nurse or the physician at the unit every second to third month with closer visits if needed, according to the patient’s glycaemic control.

Participants may report any adverse events or other unintended effects of the intervention to the facilitators or to their usual HCP. If needed, further psychological support is available in the diabetes team.

### Measures and data collection

Data will be collected shortly before randomization and at 6 and 12 months. At 6 and 12 months the questionnaires will be sent by mail to participants to fill in and to send back to JH in pre-addressed envelopes. If they fail to respond, the participants will be contacted by telephone. Baseline data will be used to describe characteristics of participants who discontinue.

The primary outcome is HbA1c at 12 months after the randomization. Secondary outcomes are self-management and psychosocial parameters based on questionnaires.

Data to be collected from participants includes: 1) HbA1c (DCA 2000), height, weight, and insulin dose per kilo body weight; 2) self-management (daily self-monitoring of blood glucose, downloaded data on self-monitoring of blood glucose (SMBG)/CGM/FGM); 3) data on socioeconomic background; 4) other diseases/disorders; and 5) number of planned and completed visits; 6) psychosocial self-efficacy (Swedish Diabetes Empowerment Scale (Swe-DES 23) [36]).

Data to be collected from participants and, if participant < 18 years, from parents includes: 1) treatment satisfaction (Diabetes Treatment Satisfaction Scale (DTSQ) Teen and Parent [37]); 2) perceived health and QoL (Check your health [7, 38], Disabkids [39]); and 3) diabetes-related family conflicts (Diabetes Family Conflict Scale (DFCS) [40]).

In this study masked assessors are not used. The primary outcome is a solid biochemical measure and the secondary outcomes include measurements on ordinal scales from validated questionnaires, and they should not be sensible to external influence. The other secondary outcomes (height, weight, insulin dose, SMBG/FGM/CGM) are registered by nurses not involved in the study.

#### Primary outcome

The primary outcome is HbA1c at 12 months after the randomization. HbA1c will be measured in connection with clinical visits right before randomization and at 6 and 12 months after. We define the baseline HbA1c as the mean HbA1c at randomization and 6 months prior to randomization in order to capture a representative value.

HbA1c levels will be assessed from capillary blood tests and analysed in the DCA Vantage apparatus (Siemens Healthcare Diagnostics, Upplands Väsby, Sweden).

#### Secondary outcomes

Data on self-management will be assessed by downloading data from self-monitoring of blood glucose (SMBG, CGM, and FGM). This way of measuring self-management has been shown to be effective in an earlier study [41]. Before randomization, each participant will fill in a questionnaire on socioeconomic data (occupation and family structure) and whether the patient has a neuropsychiatric disorder or other disease. The number of planned and completed visits will be collected from all participants by the patient record. All questionnaires that are used in this study are validated and have been used by our research group in previous studies.

Swe-DES 23 [36] will be used to measure the psychosocial self-efficacy of individuals with diabetes and has been validated in Sweden. Perceived self-efficacy is related to the willingness and the ability of people to engage in different behavioural challenges including managing a disease [42]. Swe-DES 23 measures willingness to change, self-awareness, problem identification, and stress management in a 23-item questionnaire and is sensitive to change.

We will use the DTSQ Teen and Parent [37] to measure treatment satisfaction. This instrument has recently been translated by our research group and validated to Swedish and is a widely used instrument internationally for measuring treatment satisfaction. “DTSQ Teen and Parent” measures treatment satisfaction on a 0 to 6 scale and enables comparison between the teenagers’ reports and their parents’ reports. “DTSQ Teen” is a 12-item questionnaire and “DTSQ Parent” is a 14-item questionnaire.

“Check your health” [7, 38] will be used to measure health distress and the burden of diabetes, perceived physical and emotional health, social well-being, and general QoL. It measures, on four vertical thermometer scales (0–100), perceived physical and emotional health, social well-being, and general quality of life as of now and, hypothetically, if the individual did not have diabetes. It is constructed to measure the burden of diabetes. The questionnaire can be completed in a short time and is easy to understand. We find it especially important to detect any change in the burden of diabetes since one expectation using the GSD method is to better identify and work with individual difficulties and contributing factors to glycaemic control, to increase acceptance of disease, and to decrease the burden of diabetes. To measure the generic health of children with chronic conditions, we will use the 37-item questionnaire “Disabkids” [39] that also has a specific 10-item diabetes module.

The Diabetes Family Conflict Scale (DFCS) [40] will be used to assess the level of diabetes-specific conflicts in families with children and adolescents with T1DM. The DFCS is a self-report questionnaire, and measures how frequently parents and adolescents argue about 19 tasks of diabetes management. Scores range from 19 to 57 where higher scores reflect more conflict. The importance of evaluating the degree of diabetes-related conflicts within families is emphasized since higher HbA1c has been shown to be more common in children from families with more conflicts [27].

#### Data management

Participants will be coded in the randomization process and all paper versions of coding and collected data will be stored in fireproof cabinets. Digital data will be stored in secure coded files by JH.

#### Confidentiality

Due to Swedish law (the Swedish Privacy Protection Law), data will not be available for, or shared with, other researchers.

### Statistical analysis and power calculation

To detect a difference of 6 mmol/mol in HbA1c (SD ± 10), which is a clinically relevant difference and based on previous studies [43], it is necessary to include at least 44 participants in each group (power 80% and alpha 0.05). Taking a dropout rate of 12% into consideration, a total of 100 patients will be included in the study. All analyses will be conducted on an intention-to-treat basis, which also can allow evaluation of the feasibility of the method. Differences between the groups will be analysed with un-paired Student’s *t* test with a 95% confidence interval, or non-parametric Mann-Whitney test. *P* < 0.05 will be considered as statistically significant. Multiple linear regression analysis will be used to examine which variables contribute the most to glycaemic control at 6 and 12 months.

### Data monitoring

A Data Monitoring Committee (DMC) is not used in this study. Since the study is not considered large or complex and not associated with high risk or resulting in any danger for the participants, it should not be necessary to use a DMC. There are no plans for interim analysis. The principal investigator, ALO, is responsible for ny potential decision to terminate the trial.

## Discussion

This study will evaluate the effect of the education method guided self-determination-young (GSD-Y) on glycaemic control in female adolescents with T1DM. In young females, poor glycaemic control is common and increases the risks for complications that may also have a negative impact on future pregnancy outcome. Several studies suggest that it is important to find new strategies to support young females in diabetes self-management [9, 12, 15, 44], but there are few interventions targeted at this group of patients. An acceleration of vascular complications has been described during puberty, especially in females [45], and in young adult females a higher frequency of microvascular complications compared with males has been reported [12]. In females, but not in males, illness perceptions (i.e. how you perceive and act upon your illness) and the ability to control diabetes have been found to affect the level of glycaemic control [18]. Furthermore, diabetes healthcare for adolescents should focus on identifying coping strategies since these have been shown to significantly relate to metabolic control [19]. This underlines the need for new approaches in diabetes treatment and special attention on females.

The GSD-Y method can help the patient to improve glycaemic control by offering an individualized approach based on identification of the patient’s own risk factors for later complications and by clarifying the patient’s views, perceptions, and coping strategies in managing diabetes treatment. The GSD method has previously been associated with improved glycaemic control and decreased psychosocial stress in young adult women with T1DM, but showed no significant change in glycaemic control or psychosocial measurements in men [31]. Accordingly, the method might be more favourable for female patients. In our research group, we have tested the GSD-Y method in a pilot-study on adolescent female patients with successful results. In conclusion, only female adolescents will be included in the current study, as they constitute a high-risk group for poor metabolic control.

It is a strength of our study that questionnaires used for the measurements of secondary outcomes are all validated in Swedish. Another strength of our study is that trained diabetes nurses and physicians can provide the intervention with GSD-Y without the need for referral to a psychologist or a counsellor.

The intervention will take 3 to 6 months and will be performed within the ongoing care for patients with T1DM at Sachs’ Children and Youth Hospital and at Ersta Hospital in Stockholm. Sachs’ Children and Youth Hospital is the only paediatric diabetes clinic in Sweden that cares for adolescents with T1DM up to 20 years of age. The adult diabetes outpatient clinic at Ersta Hospital has the lowest average age of T1DM patients in Sweden. Thus, these centres can provide us with enough patients for inclusion in this study. It is important to note that patients with type 1 diabetes are selected to the outpatient clinics mainly by their residency area.

It cannot be ruled out that any potential effect on HbA1c levels in this study is only a consequence of increased numbers of visits to the diabetes clinic. For patients with HbA1c above 63 mmol/mol the recommendation is more frequent visits with a diabetes nurse or a physician every 4 to 6 weeks. Accordingly, many patients in the control group will also see their caregivers more often without getting the education with GSD. To investigate the effect of the number of visits, we will register both planned and completed visits for all participants.

When seeing a patient more frequently it is sometimes difficult to get the patient’s interest and to continue forward in the process of working with problem areas. It is also common that there may be late cancellations. The GSD-Y method might increase the patient’s motivation to come to the hospital and may, together, work as a tool for both patients and HCPs in supporting diabetes self-management.

Finally, the GSD method might be seen as a time-consuming method and, if it proves successful, we plan to develop a compressed version of the method in collaboration with Assistant Professor Zoffmann who created the GSD-Y method.

### Trial status

Protocol version number 5, 26 October 2017. Recruitment began on 22 May 2017 and an approximate date when recruitment will be completed is January 2020. Any important protocol modifications will be reported to the ISRCTN Registry and the participants will be informed by the researchers.

## Additional file

Additional file 1: SPIRIT checklist. (DOC 122 kb)

## Abbreviations

BMI: Body mass index
CGM: Continuous glucose monitoring
CSII: Continuous subcutaneous insulin infusion
DFCS: Diabetes Family Conflict Scale
DMC: Data Monitoring Committee
DTSQ: Diabetes Treatment Satisfaction Questionnaire
FGM: Flash glucose monitoring
GSD: Guided self-determination
GSD-Y: Guided self-determination-young
HbA1c: Glycosylated haemoglobin
HCP: Healthcare provider
MDI: Multiple daily injections
QoL: Quality of life
SMBG: Self-monitoring of blood glucose
Swe-DES 23: Swedish Diabetes Empowerment Scale
T1DM: Type 1 diabetes mellitus
